# Infective endocarditis secondary to coagulase-negative staphylococcus revealed by endogenous endophthalmitis: Case report

**DOI:** 10.1016/j.amsu.2021.102788

**Published:** 2021-09-04

**Authors:** Hanane Choukrani, Anass Maaroufi, Boutar Ould Mohamed Sidi, Mohamed Ghali Bennouna, Abdennasser Drighil, Rachida Habbal

**Affiliations:** a Service de Cardiologie, Centre Hospitalier Universitaire Ibn Rochd, Casablanca, Morocco; b Faculté de Médecine et de Pharmacie, Université Hassan II, B.P5696, Casablanca, Morocco

**Keywords:** Endocarditis infective, Endogenous endophthalmitis, Coagulase-negative staphylococcus

## Abstract

**Introduction:**

Endogenous endophthalmitis is a rare disease which remains a diagnostic and therapeutic emergency. Infective endocarditis is the first etiology, and coagulase-negative staphylococcus is most often incriminated in endocarditis on prosthesis and rarely on native valve.

**Case report:**

We report the case of a 70 year old female patient, who has a chronic renal failure on haemodialysis, admitted to the ophthalmology department in endogenous endophthalmitis. Blood cultures, intravitreal sampling and culture of the haemodialysis catheter were positive for a multi-sensitive coagulase-negative staphylococcus. On transthoracic and transoesophageal ultrasound, vegetation was found in the right atrium along the path of the haemodialysis catheter and in the aortic and mitral valves. The patient received intravenous antibiotic and intravitreal injections of antibiotics. The evolution was marked by a systemic improvement but the ocular prognosis was poor and the evisceration decision was taken.

**Discussion:**

Endogenous endophthalmitis is related to a metastatic infectious process secondary to haematogenous microbial dissemination. The germs involved are Gram-positive bacteria and occurs very frequently in predisposed conditions. A primary infectious site is found in 90% of cases and this is most frequently endocarditis. Coagulase-negative staphylococci are responsible for 20–45% of endocarditis in prosthetic valves and the prevalence in native valve endocarditis is considered low.

**Conclusion:**

Endogenous endophthalmitis remains an emergency. The search for a source of infection, primarily endocarditis, is systematic. The improvement of the prognosis depends essentially on the eviction and early treatment of infectious foci in people at risk.

## Highlights

Endogenous endophthalmitis due to infective endocarditis is a rare disease and remains a diagnostic and therapeutic emergency.
Coagulase-negative staphylococcus is rarely incriminated in endocarditis on native valve.
Endophthalmitis is a medical emergency, due to a life-threatening disease, which visual prognosis is directly related to the time interval between the onset of the infection and its treatment.

## 1 Introduction

Endogenous endophthalmitis is a rare intraocular disease, which is secondary to haematogenous spread of germs from extraocular sites. Usually, systemic disease such as endocarditis, meningitis or urinary tract infection presents before ocular disease [1]. Endogenous endophthalmitis is a diagnostic and therapeutic emergency involving the ocular prognosis and which may be due to life-threatening diseases [[2], [3]]. We report the case of a patient admitted with endogenous endophthalmitis revealing an infective endocarditis on a coagulase-negative staphylococcus on tunnelled haemodialysis catheter, in whom the evolution after appropriate treatment was marked by a systemic improvement but a loss of the eye with the need for evisceration. This case report was reported according to SCARE criteria [4].

## 2 Case report

A 70 year old woman was admitted to the ophthalmology department with decreased visual acuity and ocular pain.

She had a history of chronic end-stage renal failure for 3 years, with polycystic kidney disease and liver disease discovered 15 years ago, haemodialysis with 2 sessions per week on a tunnelled jugular catheter that has not been changed for 2 years.

The history of her illness dated back to 1 month before her admission with the onset of generalized asthenia associated with a weight loss of 2 kg over 1 month evolving in a context of feverish sensation. Probabilistic antibiotics were prescribed without clinical improvement. The evolution was marked by the installation 1 week before his admission of an intense pain in the left eye associated with a fall of the visual acuity and a palpebral tumefaction without notion of surgery or ocular traumatism.

On admission, the patient was pale, conscious, hemodynamically and respiratorily stable, and apyretic at 37.1. Examination of the left eye revealed a 360-degree palpebral edema, corneal edema, central corneal opacity, high occular tension with a stony globe, and ophthalmoplegia.

**Fig. 1:**
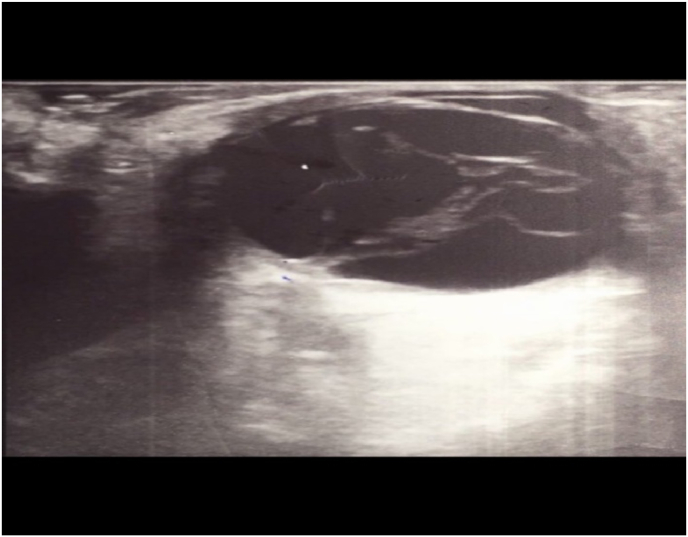
Ocular ultrasound showing a vitreous with an echogenic organization, attached to the detached temporal retinal layer.

Ocular ultrasound showed a vitreous with an echogenic organization, attached to the detached temporal retinal layer (Fig. 1).

The biological analysis showed microcytic hypochromic inflammatory anaemia at 5.6 g/dL with a ferritinemia level of 137 ng/ml, neutrophils at 10860/μL and thrombocytosis at 420,000/μL, CRP elevated to 141 mg/dL, PCT elevated to 1.22 mg/l, and rheumatoid factor elevated to 303.4 IU/ml.

**Fig. 2:**
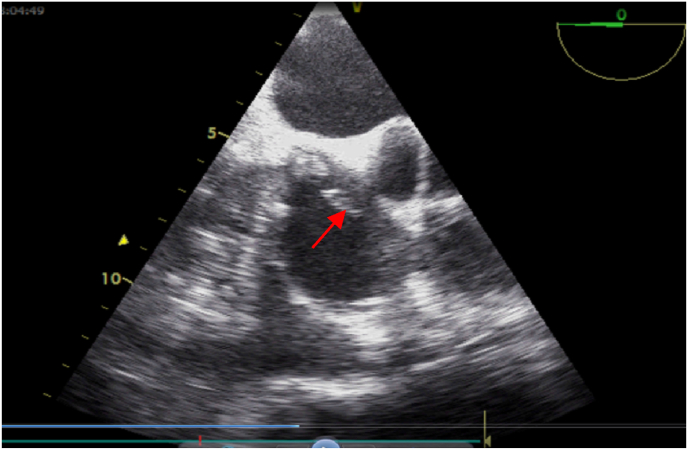
Transoesophageal echocardiography section showing vegetation at the haemodialysis catheter.

**Fig. 3:**
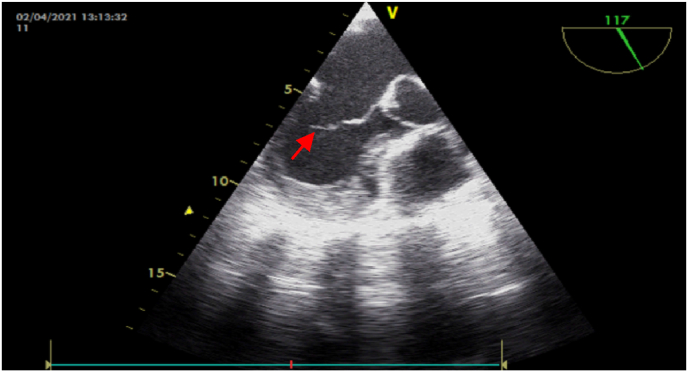
A transoesophageal echocardiography section showing vegetation at the anterior mitral valve.

As part of the etiological assessment of the endogenous endophthalmitis, a transthoracic and then transoesophageal echocardiogram were performed, on the fifth day of hospitalisation, visualizing a large vegetation measuring 15.9 × 3 mm in the right atrium on the path of the haemodialysis catheter, vibratile (Fig. 2), associated with 2 small vegetations in the right and posterior aortic cusps responsible for moderate leakage, and a small vegetation on the small mitral valve responsible for minimal leakage (Fig. 3). Intravitreal sampling and two blood cultures were positive for coagulase-negative staphylococcus. The cerebro thoracic abdominal and pelvic computed tomography did not show any other secondary location of the endocarditis.

The patient received 3 intravitreal injections of vancomycin 1 mg and ceftazidime 2 mg, and intravenous antibiotic therapy with ceftriaxone 2 g/day and gentamycin 2 mg/kg per dialysis, over a period of 4 weeks. a removal of the tunnelled catheter with a bacteriological study showing the culture of the coagulase-negative staphylococcus.

The short-term evolution was marked by clinical improvement with restoration of the general condition, biological improvement with normalisation of the infectious work-up and negativation of blood cultures and echocardiograpic improvement with regression of the size of the vegetations. Nevertheless, the ocular prognosis was poor with loss of the eyeball and a decision was made to eviscerate the eye. The patient was reviewed at 3 months after hospitalisation and evisceration was performed.

## 3 Discussion

Endophthalmitis is a medical emergency whose visual prognosis is directly related to the time interval between the onset of the infection and its treatment.

Endogenous endophthalmitis is related to a metastatic infectious process secondary to haematogenous microbial dissemination from an extraocular infection site or following intravenous administration of a contaminated solution [5].

Endogenous endophthalmitis is an exception and accounts for 2–8% of endophthalmitis [6,7]. It can affect one or both eyes, and the second eye may be affected simultaneously or at a distance [2].

The germs involved are mainly bacterial; fungal endophthalmitis is rarer. The bacteria involved are mainly Gram-positive bacteria, in order of frequency: *Staphylococcus aureus* [2], Streptococcus pneumoniae and viridans [8]. Bacterial endogenous endophthalmitis occurs very frequently in predisposed conditions (60–90% of cases [6]), most frequently diabetes [2], following invasive surgery, endoscopy, prolonged vascular catheterisation, haemodialysis, immunosuppression, cancer, systemic lupus erythematosus.

A primary infectious site is found in 90% of cases [2]. This is most frequently endocarditis (46% of cases) [2]. Exceptionally, endogenous endophthalmitis can occur in healthy individuals without risk factors.

Initial treatment includes intravitreal injection of broad-spectrum antibiotics, most commonly vancomycin and ceftazidime, and intravenous antibiotic therapy which is secondarily adapted, depending on the results of the culture and antibiogram. In severe cases, vitrectomy and intraocular corticosteroids may be indicated.

Coagulase-negative staphylococci (CNS) are responsible for 20–45% of endocarditis in prosthetic valves [11,12]. Contamination is usually peri-operative, with a majority of strains resistant to methicillin. The prevalence of SCN in native valve endocarditis is considered low. The usual percentages quoted are 1–3% [10]. However, a higher frequency was noted in some groups, notably in a large cohort of 2212 patients with infective endocarditis [9], the proportions were similar in Europe (7%) and the USA (6%). 20% had a long-term intravascular catheter and 40% had infective endocarditis associated with health care procedures.

They often complicate pre-existing valve disease and have a subacute course [13]. Embolic, cerebral or peripheral complications were reported in 15 cases (25%).

The frequencies of neurological complications according to the germs are as follows: *S. aureus* 53–65%, streptococci/enterococci: 30–36% and S. epidermidis: 14-10% [14,15]. The majority of strains are sensitive to meticillin. A reported hospital mortality rate was 19% [9].

Limitations: In our case, the haemodialysis catheter was not changed regularly or an arteriovenous fistula for dialysis was not put in place. Also, the early diagnosis of infective endocarditis before the onset of complications, and in our case of the endophthalmitis, was not carried out. These two elements would have warned the microbial proliferation and its complications.

## 4 Conclusion

Endogenous endophthalmitis remains a diagnostic and therapeutic emergency involving the functional and vital prognosis. The search for a primary focus is systematic and must necessarily include the search for infective endocarditis, which is favoured by entry points such as haemodialysis catheters as in our case. Coagulase-negative staphylococcus remains incriminated, even if more rarely, in endocarditis on native valve.

Thus, the avoidance of infective endocarditis and its complications in people at risk must be a priority by an early treatment of the infectious foci of entry, adapted change of intravenous catheters or installation of arteriovenous fistula of haemodialysis, glycaemic balance in diabetics, and the watchfulness with a diagnosis and an early therapeutic management of these endocarditis must be envisaged in these people at risk.

## Sources of funding

None.

## Patient consent

A clear and written informed consent was obtained from the patient for publication of this case report and accompanying images. A copy of the written consent is available for review by the editor in chief of this journal on request.

## Ethical approval

Not aplicable.

## Author contribution

CHOUKRANI Hanane, corresponding author, writing the paper.

## Research registration

None.

## Guarantor

CHOUKRANI Hanane.

## Provenance and peer review

Not commissioned, externally peer-reviewed.

## Author contribution

CHOUKANI Hanane: corresponding author, data collection, writing the paper and making the revisions following the reviewer's instructions.MAAROUFI Anass: concept and writing the paper.OULD MOHAMED SIDI Boutar: data collection.BENNOUNA Mohamed Ghali: reviewing.DRIGHIL Abdennasser: reviewing.HABBAL Rachida: reviewing and validating.

## Registration of research studies

Name of the registry:

Unique Identifying number or registration ID:

Hyperlink to your specific registration (must be publicly accessible and will be checked):

## Guarantor

CHOUKRANI Hanane.

## Consent

A clear and written informed consent was obtained from the patient for publication of this case report and accompanying images. A copy of the written consent is available for review by the editor in chief of this journal on request.

## Declaration of competing interest

Authors of this article declare having no conflict or computing interest.
